# 5-Methoxyflavone alleviates LPS-mediated lung injury by promoting Nrf2-mediated the suppression of NOX4/TLR4 axis in bronchial epithelial cells and M1 polarization in macrophages

**DOI:** 10.1186/s12950-022-00319-6

**Published:** 2022-11-30

**Authors:** Panqiao Liang, Linxin Wang, Sushan Yang, Xiping Pan, Jiashun Li, Yuehan Zhang, Yueyun Liang, Jing Li, Beixian Zhou

**Affiliations:** 1 Center of Stem Cell and Regenerative Medicine, The People’s Hospital of Gaozhou, Gaozhou, 525200, China; 2 Department of Clinical Medicine, The Third Clinical School of Guangzhou Medical University, Guangzhou Medical University, Guangzhou, 511436 China; 3 Guangzhou Laboratory, Guangzhou, China; 4 Department of Clinical Laboratory, The People’s Hospital of Gaozhou, Gaozhou, 525200 China; 5 Department of Respiratory, Affiliated Huadu Hospital, Southern Medical University (People’s Hospital of Huadu District), Huadu, 510800 China; 6 Department of Anesthesiology, The People’s Hospital of Gaozhou, Gaozhou, 525200 China; 7 State Key Laboratory of Respiratory Disease, National Clinical Research Center of Respiratory Disease, Guangzhou Institute of Respiratory Health, the First Affiliated Hospital of Guangzhou Medical University, Guangzhou Medical University, Guangzhou, China; 8 Institute of Chinese Integrative Medicine, Guangzhou Medical University, Guangzhou, Guangdong China

**Keywords:** Methylated flavonoid, 5-Methoxyflavone, Nrf2, LPS, Acute lung injury

## Abstract

**Background:**

Acute lung injury (ALI) arises from sepsis or bacterial infection, which are life-threatening respiratory disorders that cause the leading cause of death worldwide. 5-Methoxyflavone, a methylated flavonoid, is gaining increased attention for its various health benefits. In the current study, we investigated the potential effects of 5-methoxyflavone against LPS-mediated ALI and elucidated the corresponding possible mechanism.

**Methods:**

A mouse model with ALI was established by intratracheal instillation of LPS, and lung pathological changes, signaling pathway related proteins and apoptosis in lung tissues were estimated by H&E staining, immunofluorescence and TUNEL assay, respectively. Cell viability was evaluated by MTT assay; protein levels of pro-inflammatory mediators were measured by ELISA assay; levels of ROS and M1 macrophage polarization were assayed by flow cytometry; the expression of Nrf2 signaling, NOX4/TLR4 axis and P-STAT1 were detected by western blotting.

**Results:**

Our results showed that 5-methoxyflavone treatment inhibited LPS-induced expression of NOX4 and TLR4 as well as the activation of downstream signaling (NF-κB and P38 MAPK), which was accompanied by markedly decreased ROS levels and pro-inflammatory cytokines (IL-6, TNF-α, MCP-1, and IL-8) in BEAS-2B cells. Moreover, we revealed that these effects of 5-methoxyflavone were related to its Nrf2 activating property, and blockade of Nrf2 prevented its inhibitory effects on NOX4/TLR4/NF-κB/P38 MAPK signaling, thus abrogating the anti-inflammatory effects of 5-methoxyflavone. Besides, the Nrf2 activating property of 5-methoxyflavone in RAW264.7 cells led to inhibition of LPS/IFN-γ-mediated STAT1 signaling, resulting in suppression of LPS/IFN-γ-induced M1 macrophage polarization and the repolarization of M2 macrophages to M1. In a mouse model of LPS-induced ALI, 5-methoxyflavone administration ameliorated LPS-mediated lung pathological changes, the increased lung index (lung/body weight ratio), and epithelial cell apoptosis. Meanwhile, we found 5-methoxyflavone effectively suppressed the hyperactive signaling pathways and the production of excessive pro-inflammatory mediators. Moreover, 5-methoxyflavone reduced LPS-mediated M1 macrophage polarization associated with elevated P-STAT1 activation in the lung tissues. In addition, 5-methoxyflavone improved the survival of LPS-challenged mice.

**Conclusion:**

These results indicated that 5-methoxyflavone might be suitable for the development of a novel drug for ALI therapeutic.

## Background

Acute lung injury (ALI) and its more severe form, acute respiratory distress syndrome (ARDS), are life-threatening respiratory disorders that pose a substantial threat to mankind’s health worldwide [1]. Among various etiologies (e.g., trauma, shock, sepsis) that contribute to ALI, the respiratory system with gram-negative bacteria exposure is the most common risk factor [2]. Pathologically, bacteria-associated lung injury is characterized by non-cardiogenic pulmonary edema, neutrophil accumulation, extensive alveolar damage, and sustained excessive pulmonary inflammation. Despite advances in supportive care and antibacterial agents, the incidence and mortality of ALI/ARDS are still high, ranging from 25 to 45% [2–4]. Currently, there is still no specific therapeutic strategy for ALI/ARDS treatment, which highlights the necessity of continuous effort to develop novel approaches or agents for ALI/ARDS therapy.

It is well-established that initiation of the pro-inflammatory response is the result of the interaction between pattern recognition receptors (PRRs) and conserved structures from microorganisms, called pathogen-associated molecular patterns (PAMPs) [5]. As one of the most extensively studied PAMPs, lipopolysaccharide (LPS) is a main virulence factor derived from the outer envelope of gram-negative bacteria. TLR4, a cell-surface PRR that is ubiquitously expressed in structural lung cells, specifically recognizes LPS, leading to elevation of reactive oxygen species (ROS) and triggering activation of downstream signaling cascades, such as NF-κB and MAPK signalings [6]. Upon LPS stimulation, the increased expression of cytokines and chemokines, such as IL-6, TNF-α, RANTES and IL-8, is believed to be dependent on these signals [5]. Simultaneously, macrophages and neutrophils attracted by the released chemokines migrate from the bloodstream to the site of infection [5]. It is noteworthy that the appropriate levels of ROS and inflammation play a crucial role in the induction of innate immunity and promotion of pathogen clearance [7]. While, it has been reported that uncontrolled ROS production, excessive pro-inflammatory mediators and increased innate immune cell influx play a critical role in the pathogenesis of LPS- or gram-negative bacteria-triggered ALI [6]. The integrity of the lung vascular endothelial barrier could protect against LPS-induced lung injury [6]. Evidence has been demonstrated that the levels of tight junction-associated proteins (e.g., Occludin ZO-1 and Claudin-5) were reduced by ROS and pro-inflammatory mediators (IL-8 and TNF-α), resulting in enhanced lung vascular endothelial cell permeability and leakage, followed by extravasation of blood components (plasma, macromolecules and blood cells) [6, 8]. In response to an inflammatory microenvironment, the recruited macrophages in the lung have a polarized M1/M2 phenotype and exhibit proinflammatory and anti-inflammatory properties, respectively [9]. It has been found that M1 macrophages impaired endothelial barrier function, and promoted apoptosis of endothelial cells and alveolar epithelial cells during LPS challenge [10]. Based on these accumulating evidences, it is conceivable that attenuation of excessive pro-inflammatory response may exert protective effects against gram-negative bacteria- or LPS-induced lung injury.

Upon exogenous stimulations (such as oxidative and xenobiotic stresses), the pleiotropic transcription factor Nrf2 (Nuclear factor erythroid-2-related factor 2) dissociated from its repressor KEAP1 (Kelch-like ECH-associated protein 1) that translocates into the nucleus where it binds to the antioxidant response elements (AREs), and drives expression of detoxification and cytoprotective enzymes, such as heme oxygenase-1 (HO-1), NAD(P)H quinone oxidoreductase-1 (NQO1) and glutathione S-transferases (GSTs) [11]. Activation of Nrf2 signaling has been demonstrated to provide beneficial effects in the treatment of inflammatory diseases, including sepsis, viral infections and asthma [12–14]. While, knockout of Nrf2 in mice resulted in exacerbated inflammation conditions that contributed to adverse outcome of sepsis and emphysema [15, 16]. Consistently, Nrf2 activation in immune cells could also alleviate various PAMPs (e.g., LPS, viral RNA) -triggered inflammation [17]. Nrf2-based treatment can act as an attractive target for various inflammation disease controlling. It should be noted that the level of Nrf2 is low under normal physiological conditions [18]. It is therefore proposed that properly timed modulation of Nrf2 activation by compounds may provide therapeutic potential for gram-negative bacteria- or LPS-associated lung injury.

Naturally occurring flavonoids are rich in fruits, vegetables and herbs, which possess various kinds of biological activities, including anti-inflammatory [19], anti-oxidant [20], anti-viral [21] and anti-tumor [22]. Interestingly, the bioactivity and bioavailability of methylated flavones have been reported to be superior to those of unmethylated flavones [23]. Among methylated flavonoids, 5-methoxyflavone with improved hepatic metabolic stability and intestinal absorption was reported [24]. And 5-methoxyflavone exhibited an anti-tumor effect via induction of apoptosis and autophagy [25]. However, the protective effects and corresponding possible mechanisms of 5-methoxyflavone on LPS-induced inflammatory diseases remain poorly understood. Therefore, the purpose of our study was to investigate the effects of 5-methoxyflavone on LPS-mediated in vitro and in vivo and their related mechanisms.

## Materials and methods

### Regents

**Fig. 1:**
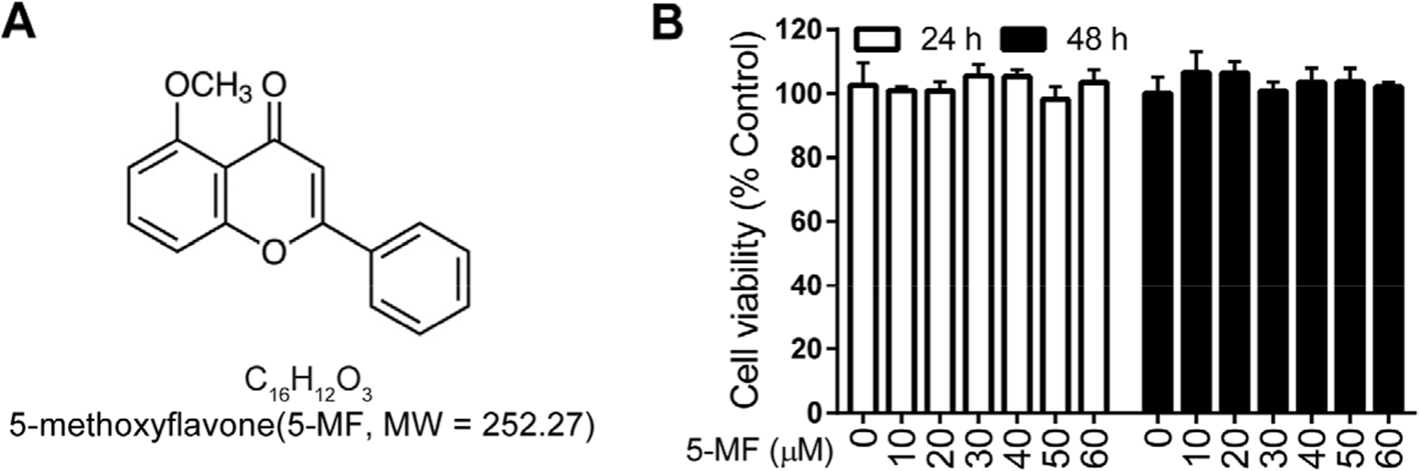
Effects of 5-methoxyflavone on the viability of BEAS-2B cells. (**A**) Chemical structure of 5-methoxyflavone (5-MF). (**B**) BEAS-2B cells were treated with 5-methoxyflavone for 24 and 48 h. The viability of BEAS-2B cells was determined by the MTT assay.

5-Methoxyflavone (CAS-No. 42079-78-7, purity > 98.0 %) (Fig. 1A) was purchased from TCI (Tokyo Chemical Industry, Tokyo, Japan) and prepared 50 mM stock solutions in dimethyl sulfoxide (DMSO). LPS (*Escherichia coli* O111: B4) was acquired from Sigma-Aldrich (St. Louis, MO, USA). H2DCFDA (DCFH-DA, 2',7'-Dichlorodihydrofluorescein diacetate) was from MedChem Express (Monmouth Junction, NJ, USA). Primary antibodies against NOX4, TLR4, F4/80, iNOS, HO-1 and Nrf2 were purchased from Proteintech (Rosemont, IL, USA). Primary antibodies against phosphorylated-P65 (S536), P65, phosphorylated-P38 (T180/Y182), P38, phosphorylated-STAT1 (T701) and STAT1 were obtained from Cell Signaling Technology (Danvers, MA, USA). Primary antibodies against GAPDH, EpCAM, IL-6 and TNF-α were from Affinity Biosciences (Wuhan, China). The horseradish peroxidase (HRP)-conjugated goat anti-rabbit secondary antibody was obtained from Multisciences Biotech Co., Ltd. (Hangzhou, China). Recombinant mouse IFN-γ and IL-4 were purchased from Peprotech. Human ELISA kits (IL-6, TNF-α, IL-8 and MCP-1) were from R&D Systems. Mouse ELISA kits for IL-6, TNF-α and MCP-1 determination were from Multiscience (Hangzhou, China).

### Animal experiments

C57BL/6 mice (weight, 18-20g; 4-6-weeks old) were obtained from Guangdong Medical Laboratory Animal Center (Guangzhou, China) and housed in a specific pathogen-free environment. They were randomly divided into four groups: control group; LPS group; 5-methoxyflavone low dose treatment group, 5-methoxyflavone high dose treatment group. Mice were intragastrically administered with 5-methoxyflavone (25 or 50 mg⋅kg^-1^⋅d^-1^) 2 days prior to the LPS challenge. To establish ALI model, mice were anesthetized intraperitoneally with 5% chloral hydrate and intratracheally instilled with LPS (5 mg/kg) dissolved in 50 μL sterile saline. All animal experiments were strictly conducted following the guidance for the Care and Use of Laboratory Animals of the National Institutes of Health. All procedures were approved by the Animal Ethics Committee of Guangzhou Medical University.

### Cell lines and culture

Human bronchial epithelial cells (BEAS-2B) and a mouse monocytic cell line (RAW264.7) were obtained from American Type Culture Collection (ATCC, Manassas, VA, USA). Dulbecco’s modified Eagle’s medium (DMEM) containing 10% fetal bovine serum (FBS; Thermo Fisher) and 1% penicillin-streptomycin was used for BEAS-2B cells and RAW264.7 cells cultivation. The cells were maintained at 37 °C in a humidified incubator with 5% CO_2_.

### Cell viability

3-(4,5-dimethylthiazol-2-yl)-2,5-diphenyltetrazolium bromide (MTT) assay was employed for determination the cytotoxicity of 5-methoxyflavone. Briefly, cells were seeded at a concentration of 2 × 10^4^ cells/well into 96-well culture plates and cultivated at 37°C for 12 h, followed by incubation with two-fold serial dilutions of 5-methoxyflavone (0 - 60 μM) for 24 and 48 h. Then, cells were incubated with 100 μL of MTT working solution (5 mg/mL) for 4 h. Afterward, DMSO (100 μL) was added to dissolve the formazan crystals. Absorbance at 570 nm was determined by a microplate reader (Thermo Fisher Scientific, Waltham, MA, USA).

### Western blot analysis

After the indicated treatment, BEAS-2B cells and RAW264.7 cells were rinsed twice with cold PBS and lysed in ice-cold radio-immunoprecipitation (RIPA) buffer (Beyotime Biotech, Shanghai, China). After the cellular crude extracts were centrifuged at 13, 000 × g for 10 min at 4°C, the supernatants were harvested for total protein concentration quantification by using a BCA protein assay kit (Thermo Fisher Scientific, Waltham, MA, USA). Equivalent amounts of cell lysates (20 μg/lane) were electrophoresed and transferred to PVDF membrane (Bio-rad, Hercules, CA, USA). The membranes were blocked with 5% skimmed milk dissolved in 1 × TBST at room temperature for 2 h, then incubated with indicated primary antibodies at 4°C overnight. After washing three times with 1 × TBST, the membranes were incubated with horseradish peroxidase (HRP)-conjugated secondary antibodies at room temperature for 1 h. Proteins of interest were visualized by using an ECL detection kit (Amersham; GE Healthcare Life Sciences, Chalfont, UK). The band intensity of targeted proteins was determined by ImageJ software and was normalized to the intensity of GAPDH.

### Hematoxylin-eosin (H&E) staining

Mice were sacrificed under anesthesia with 5% chloral hydrate (5 mL/kg, i.p.) and lung tissues were excised and processed for fixation in 4% paraformaldehyde. Following embedded in paraffin, lung tissues were cut into 4-μm thick sections for H&E staining. Lung injury was scored in a blinded manner based on the following histological changes: hemorrhage, interstitial and alveolar edema, inflammatory infiltration, and the thickness of alveolar septum. And the scores were assigned, ranging from 0 to 4 (0, absent; 1, mild; 2, moderate; 3, severe; 4, very severe).

### Reactive oxygen species (ROS) assay

Levels of intracellular ROS were measured using the oxidant-sensing probe DCFH-DA. Briefly, BEAS-2B cells grown in six-well culture plates were exposed to LPS stimulation with or without 5-methoxyflavone treatment. After 24 h, cells were incubated with 5 μM DCFH-DA diluted in PBS at 37°C in the dark. After 30 min incubation, cells were washed three times with PBS and determined under the fluorescence microscope (Leica, Wetzlar, Germany) and Accuri C6 flow cytometry (BD Biosciences; San Jose, CA, USA).

### Pro-inflammatory mediator measurement

BEAS-2B cells were pre-treated with 5-methoxyflavone for 2 h, and then exposed to LPS. After 24 h, culture supernatants were harvested and centrifuged at 13, 000 × g for 15 min at 4°C to remove cellular debris. Aliquots of the culture supernatant were stored at -80°C. The concentrations of cytokines and chemokines in the culture supernatant were determined by ELISA assay according to the manufacturer’s protocol.

### Flow Cytometry

For phenotyping, RAW264.7 cells were collected and washed with PBS, and subsequently incubated with FITC-conjugated goat anti-mouse F4/80, PE-Cy7-conjugated goat anti-mouse iNOS and APC-conjugated goat anti-mouse CD206 (Biolegend) for 30 min at room temperature. After washing, the cells were subjected to analysis by flow cytometry with a FACScalibur (BD Biosciences).

### Immunofluorescence

BEAS-2B cells and RAW264.7 cells grown on glass coverslips in 48-well plates were treated as indicated, fixed with 4% paraformaldehyde for 15 min, and washed three times with PBS. After permeabilization with 0.5% Triton X-100, cells were blocked with 5% BSA in PBS for 30 min at room temperature. Cells were incubated with the indicated primary antibodies at 4°C overnight and incubated with fluorescein-conjugated anti-rabbit secondary antibody (multi-sciences, Hangzhou, China) for 1 h at room temperature. Nuclei were counterstained with 4’,6-diamidino-2-phenylindole (DAPI) (Beyotime, Shanghai, China). Images were captured using a Leica Stellaris confocal microscope (Leica, Wetzlar, Germany).

For immunofluorescence staining of tissue sections, paraffin-embedded lung sections were dewaxed in xylene and rehydrated in a series of gradient ethanols. Then, sections were subjected to antigen retrieval in citrate buffer (pH 6.0) in a microwave, followed by quenching of endogenous peroxidases and blocking in 10% goat serum for 30 min. The procedures for antibody incubation and nuclei staining were the same as those for immunofluorescence staining of cells. For three-color immunofluorescence, sections were stained by using a multiple immunofluorescence staining kit (G1236, Servicebio, Wuhan, China) based on the Tyramide signal amplification (TSA) technique according to the manufacturer’s manual.

### TUNEL assay

For detection of the apoptotic lung epithelial cells within the lungs, a three-color labeling technique was used. In brief, lung sections were first incubated with antibodies to EpCAM to label lung epithelial cells, and active-caspase 3 to reveal the occurrence of apoptosis. Then, DNA fragmentation was detected using a TUNEL assay commercial kit (Elabscience, Wuhan, China) according to the protocols of the manufacturer.

### Statistical analysis

All data in this study are presented as the mean ± SD and were analyzed by one-way analysis of variance (ANOVA) followed Tukey’s post-hoc analysis with the GraphPad Prism 7.0 software (Prism, La Jolla, CA, USA). p < 0.05 was considered statistically significant.

## Results

### Cytotoxicity of 5-methoxyflavone on BEAS-2B cells

In order to avoid the cytotoxicity, we first investigated the effect of 5-methoxyflavone on the cell viability of BEAS-2B cells. BEAS-2B cells were incubated with 5-methoxyflavone at increased concentrations ranging from 0 to 60 μM for 24 and 48 h, and the cell viability were evaluated by MTT assay. Compared to the untreated group, 5-methoxyflavone at concentrations up to 60 μM did not significantly affect the cell viability of BEAS-2B cells, suggesting that 5-methoxyflavone had no cytotoxic effects on BEAS-2B cells at a concentration of 60 μM (Fig. 1B). Thus, the concentrations of 5-methoxyflavone lower than 60 μM were chosen for the subsequent experiments.

### 5-Methoxyflavone suppressed the activation of TLR4/NF-κB/P38 MAPK in LPS-stimulated BEAS-2B cells

**Fig. 2:**
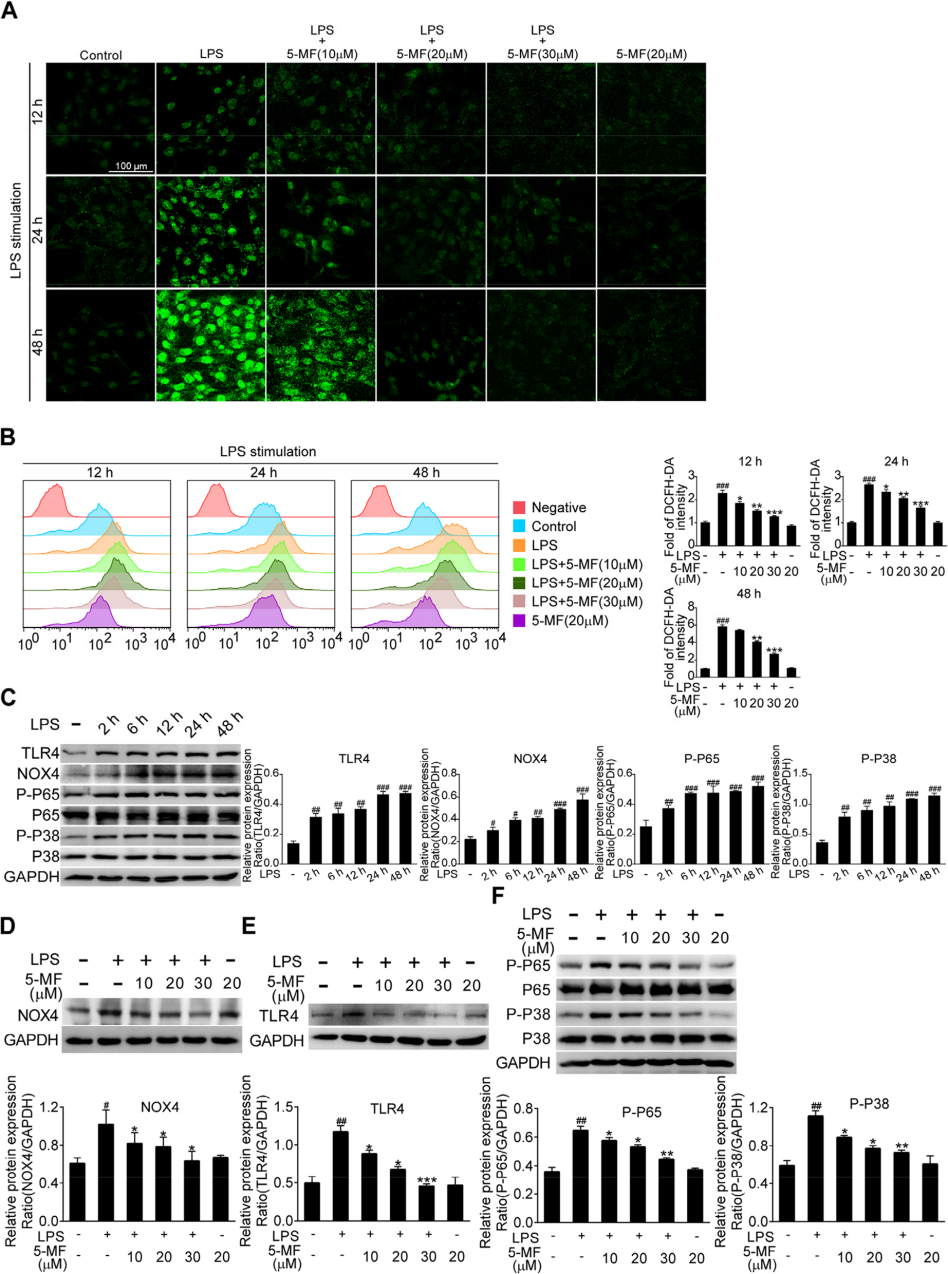
Effects of 5-methoxyflavone on the LPS-mediated NOX4/TLR4/NF-κB/P38 MAPK pathway in BEAS-2B cells. (**A**, **B**) BEAS-2B cells were stimulated with LPS (10 μg/mL) in the presence or absence of 5-methoxyflavone for 12, 24 and 48 h. Then, cells were incubated with 5 μM DCFH-DA at 37°C for 30 min. The levels of ROS in LPS-stimulated BEAS-2B cells treated with 5-methoxyflavone were detected by fluorescence microscopy (A) and flow cytometry (B). (**C**) BEAS-2B cells were stimulated with LPS (10 μg/mL) for 2, 6, 12, 24 and 48 h. Expression of NOX4, TLR4, P-P65 and P-P38 was detected by western blotting. (**D**, **E**) Expression of NOX4 (D) and TLR4 (E) in LPS-stimulated BEAS-2B cells treated with 5-methoxyflavone was detected by western blotting. (**F**) Expression of P-P65 and P-P38 in LPS-stimulated BEAS-2B cells treated with 5-methoxyflavone were detected by western blotting. The band intensity of targeted proteins was determined by ImageJ software and was normalized to the intensity of GAPDH. ^#^*p* < 0.05, ^##^*p* < 0.01, ^###^*p* < 0.001 versus the control group; ^*^p < 0.05, ^**^p < 0.01 ^***^p < 0.001 versus the LPS group

It has been reported that the release of large amounts of ROS triggered by LPS exposure could lead to excessive inflammation production and even accelerate the progression of ALI [26]. We thus investigated the effects of 5-methoxyflavone on LPS-mediated accumulation of ROS in BEAS-2B cells at 12 - 48 h using DCFH-DA staining. The results of fluorescence microscope and flow cytometry showed that BEAS-2B cells with LPS stimulation triggered the generation of ROS in time-dependent manner, which were dose-dependently reduced by 5-methoxyflavone treatment (Fig. 2A and B). It has been reported that NOX4/TLR4 signal axis was significantly activated in endothelial cells upon LPS stimulation [23]. We therefore investigated whether LPS stimulation could triggered the activation of NOX4/TLR4 signal axis in bronchial epithelial cells. As shown in Fig. 2C, LPS stimulation time-dependently increased the expression of TLR4, NOX4, P-P65 and P-P38 in BEAS-2B cells. Given that NOX4 plays a critical role in LPS-induced ROS production, we continued to investigate whether the attenuation of LPS-induced ROS was due to the inhibition of NOX4 expression by 5-methoxyflavone. Indeed, 5-methoxyflavone treatment effectively decreased the increased expression of NOX4 in LPS-stimulated BEAS-2B cells (Fig. 2D). It is reported that the interaction between NOX4 and TLR4 is essential for ROS production, the signal transduction from the cell surface and then pro-inflammatory response initiation [27]. Therefore, we next investigated whether 5-methoxyflavone affected the expression of TLR4 and the activation of downstream signaling. As expected, western blotting results demonstrated that LPS-induced the upregulation of TLR4 expression in BEAS-2B cells was significantly decreased by 5-methoxyflavone treatment (Fig. 2E). Accordingly, the activation of TLR4 downstream signaling pathways, NF-κB and P38 MAPK, was significantly inhibited by 5-methoxyflavone (Fig. 2F). Therefore, these results suggested that 5-methoxyflavone could inhibit LPS-mediated activation of NOX4/ROS/TLR4/NF-κB/P38 MAPK signaling in BEAS-2B cells.

### 5-Methoxyflavone treatment triggered the activation of Nrf2/HO-1 signaling in LPS-stimulated BEAS-2B cells

**Fig. 3:**
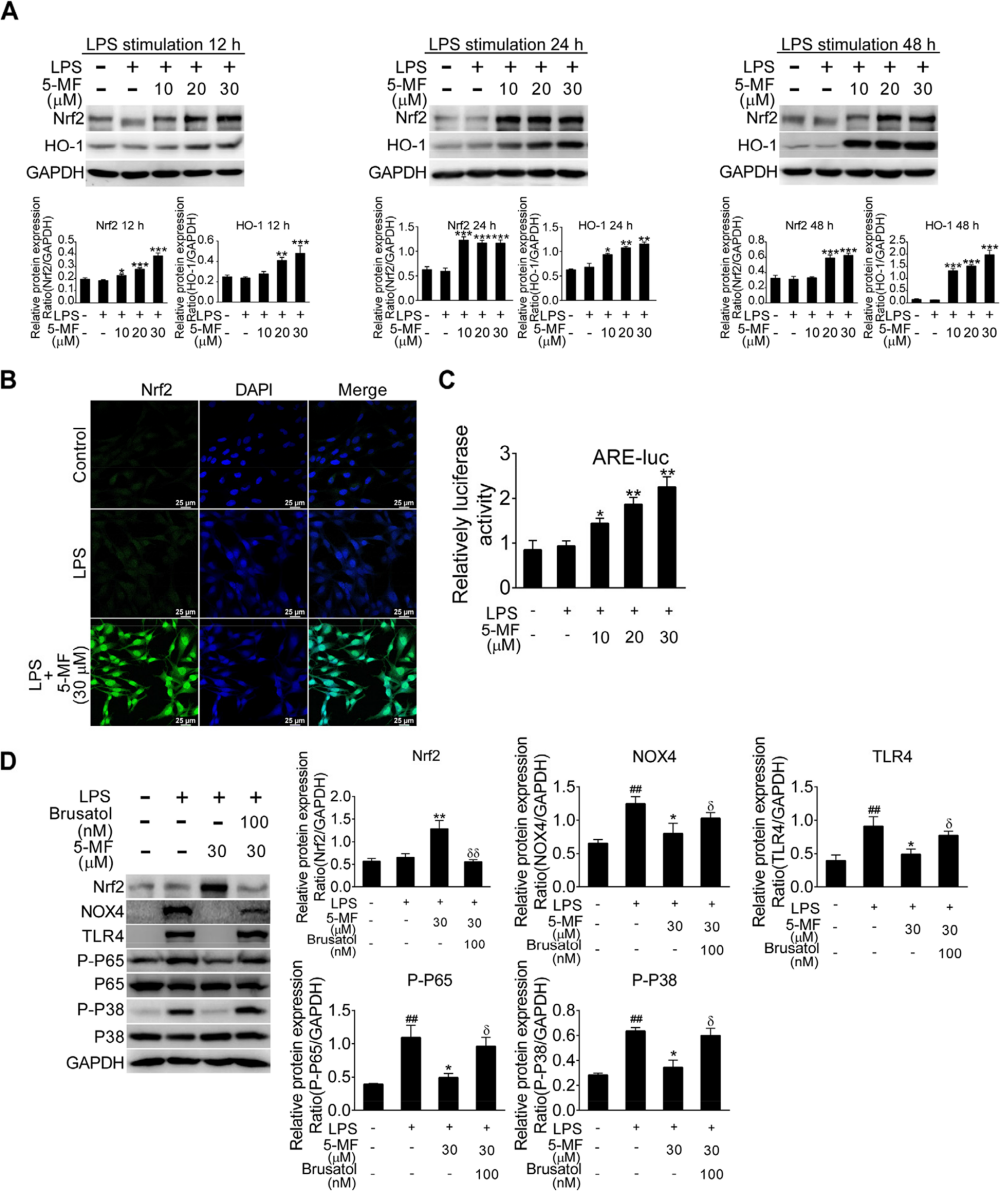
Effects of 5-methoxyflavone on the activation of Nrf2/HO-1 pathway in LPS-stimulated BEAS-2B cells. (**A**) Expression of Nrf2 pathway related proteins (Nrf2, HO-1) was detected by western blotting in LPS-stimulated BEAS-2B cells treated with 5-methoxyflavone for 12, 24 and 48 h. (**B**) Immunofluorescence analysis of 5-methoxyflavone-mediated Nrf2 nuclear translocation in LPS-stimulated BEAS-2B cells. (**C**) 5-Methoxyflavone activated the transcriptional activity of Nrf2 in LPS-stimulated BEAS-2B cells. BEAS-2B cells transfected with the pARE-Luc plasmid were treated with the indicated concentration of 5-methoxyflavone for 24 h, and then lysed for firefly and renilla luciferase activity measurement. Data was expressed as the ratio of firefly to renilla luciferase activity (Fluc/Rluc). (**D**) Expression of Nrf2, NOX4, TLR4, P-P65 and P-P38 was detected by western blotting in LPS-stimulated BEAS-2B cells treated with 5-methoxyflavone alone or in combination with brusatol treatment for 24 h. The band intensity of targeted proteins was determined by ImageJ software and was normalized to the intensity of GAPDH. ^##^*P* < 0.01 versus the control group; ^*^P < 0.05, ^**^P < 0.01, ^***^P < 0.001 versus the LPS group; ^δ^*P* < 0.05, ^δδ^*P* < 0.01 versus the 5-methoxyflavone treatment alone

Nrf2 signaling plays a protective role against various kinds of stimulus-triggered oxidant stress and inflammation [28]. Therefore, we determined to figure out whether the inhibitory effect of 5-methoxyflavone on LPS-activated NOX4/ROS/TLR4/NF-κB/P38 MAPK signaling was associated with Nrf2 signaling activation in BEAS-2B cells. As shown in Fig. 3A, LPS-stimulated BEAS-2B cells with 5-methoxyflavone treatment for 12, 24 and 48 h were found to significantly up-regulate the expression of Nrf2 signaling-associated molecules, including Nrf2 and HO-1. Meanwhile, in contrast to the weak nuclear accumulation of Nrf2 in the LPS alone group, immunofluorescence staining showed that treatment with 5-methoxyflavone dramatically increased the nuclear translocation of Nrf2 (Fig. 3B). To investigate whether the promotion of Nrf2 nuclear translocation by 5-methoxyflavone is functional, BEAS-2B cells transfected with a pARE-luc reporter plasmid containing Nrf2 binding sites were stimulated with LPS in the presence or absence of 5-methoxyflavone for 24 h. As expected, 5-methoxyflavone treatment significantly increased Nrf2-dependent reporter activity (Fig. 3C), indicating that 5-methoxyflavone could drive the transcriptional activity of Nrf2. To determine whether the suppressive effects on LPS-induced NOX4/ROS/TLR4/NF-κB/P38 MAPK signaling were attributed to Nrf2 activation, BEAS-2B cells were pre-treated with brusatol (an Nrf2 inhibitor) for 30 min prior to incubation with 5-methoxyflavone. Our results showed 5-methoxyflavone decreased LPS-upregulated expression of NOX4, TLR4, P-P65 and P-P38 that was reversed by brusatol (Fig. 3D). Taken together, these data indicated that 5-methoxyflavone treatment mediated activation of Nrf2 signaling, which contributed to the inhibition of LPS-induced ROS production and TLR4/NF-κB/P-P38 signal transduction.

### 5-Methoxyflavone inhibited LPS-mediated pro-inflammatory response via activation of Nrf2/HO-1 signaling

**Fig. 4:**
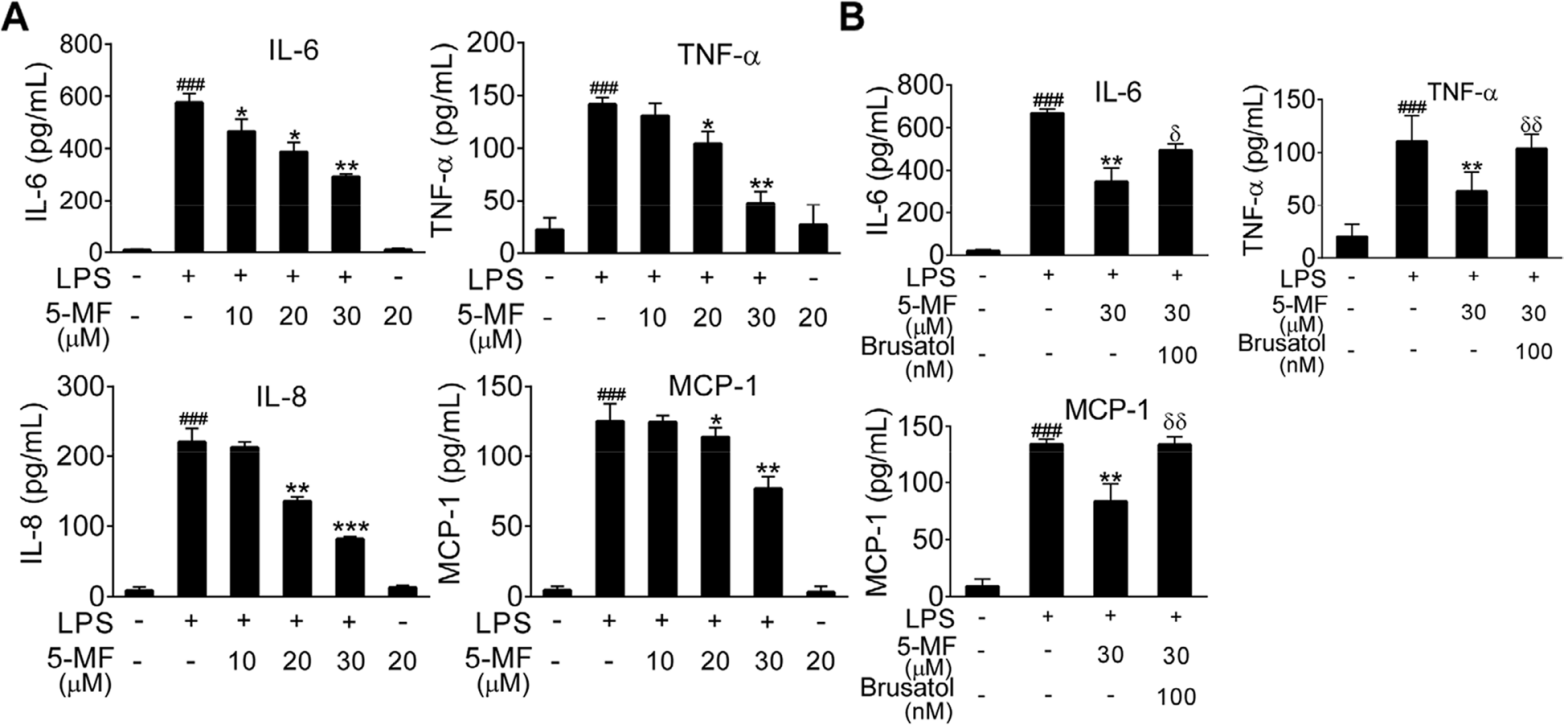
Effects of 5-methoxyflavone on LPS-mediated expression of pro-inflammatory cytokines in BEAS-2B cells. (**A**) ELISA assay was performed to determine the expression levels of IL-6, TNF-α, IL-8 and MCP-1 in the culture supernatant of LPS-stimulated BEAS-2B cells with or without 5-methoxyflavone treatment. (**B**) ELISA assay was performed to determine the expression levels of IL-6, TNF-α and MCP-1 in the culture supernatant of LPS-stimulated BEAS-2B cells treated with 5-methoxyflavone alone or in combination with Nrf2 inhibitor (brusatol). ^###^*P* < 0.001 versus the control group; ^*^P < 0.05, ^**^P < 0.01, ^***^P < 0.001 versus the LPS group; ^δ^*P* < 0.05, ^δδ^*P* < 0.01 versus the 5-methoxyflavone treatment alone

Uncontrolled pro-inflammatory mediator production has been suggested to play a crucial role in the pathogenesis of LPS-induced ALI [6]. To determine whether 5-methoxyflavone could modulate LPS-triggered excessive inflammation, we analyzed the levels of cytokines in the culture supernatant of LPS-stimulated BEAS-2B cells. As expected, the increased levels of pro-inflammatory cytokines and chemokines, including IL-6, TNF-α, IL-8 and MCP-1, were decreased in LPS-stimulated BEAS-2B cells with 5-methoxyflavone incubation (Fig. 4A). Since 5-methoxyflavone could activate Nrf2 signaling, we then investigated whether the suppression of LPS-mediated inflammation by 5-methoxyflavone was correlated with Nrf2 activation. And we found that the combination of 5-methoxyflavone with brusatol impaired the inhibitory effects of 5-methoxyflavone on the expression of these pro-inflammatory mediators (Fig. 4B). Therefore, these results indicated that 5-methoxyflavone treatment reduced LPS-mediated inflammation via activation of Nrf2 signaling.

### 5-Methoxyflavone reversed M1-like polarization of RAW264.7 cells

**Fig. 5:**
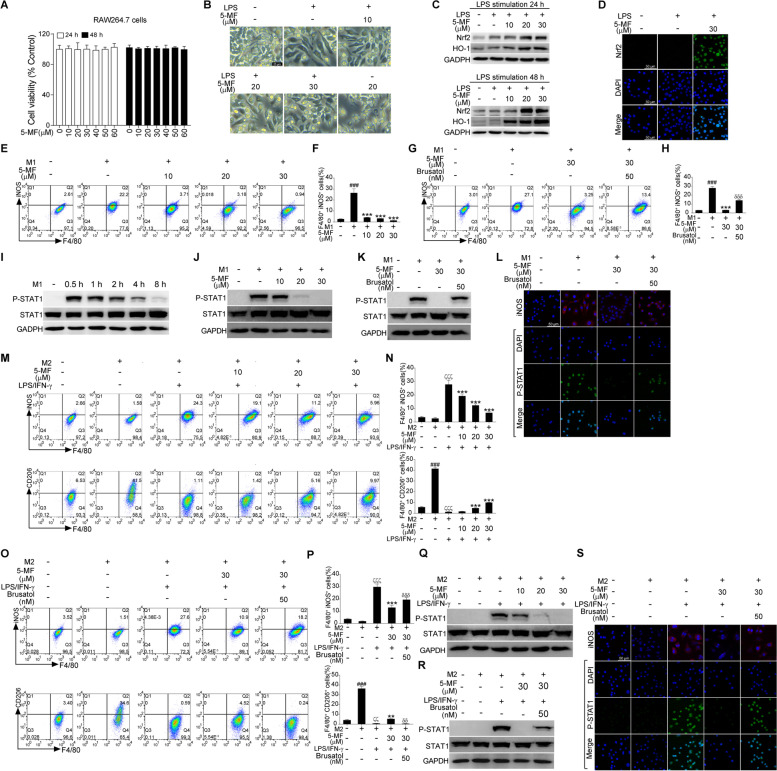
Effects of 5-methoxyflavone on RAW264.7 cells M1 polarization and M2 repolarization. (**A**) RAW264.7 cells were treated with 5-methoxyflavone for 24 and 48 h. The viability of RAW264.7 cells was determined by the MTT assay. (**B**) Representative images of morphological changes of LPS-stimulated RAW264.7 cells with or without 5-methoxyflavone treatment were obtained under a microscope. (**C**) RAW264.7 cells were treated with LPS (100 ng/mL) in the presence or absence of 5-methoxyflavone for 24 and 48 h. Expression of Nrf2 and HO-1 was detected by western blotting. (**D**) Immunofluorescence analysis of 5-methoxyflavone-mediated Nrf2 nuclear translocation in LPS-stimulated RAW264.7 cells. (**E**-**H**) RAW264.7 cells were stimulated with a combination of LPS (100 ng/mL) plus IFN-γ (20 ng/mL) for M1 polarization and IL-4 (20 ng/mL) for M2 polarization. Flow cytometry analysis of M1 marker (iNOS) in LPS/IFN-γ-stimulated RAW264.7 cells with indicated concentration of 5-methoxyflavone treatment alone (E and F) or with combinations of 5-methoxyflavone plus brusatol treatment (G and H) for 24 h. ^###^*P* < 0.001 versus the control group; ^***^P < 0.001 versus the M1-polarized RAW264.7 cell group; ^δδδ^P < 0.001 versus the 5-methoxyflavone treatment alone. (**I**) After stimulation with LPS/IFN-γ for 0.5 - 8 h, RAW264.7 cells were lysed for analysis of the activation of P-STAT1. (**J**, **K**) Western blot analysis of P-STAT1 in LPS/IFN-γ-stimulated RAW264.7 cells with indicated concentration of 5-methoxyflavone treatment alone (J) or with combinations of 5-methoxyflavone plus brusatol treatment (K) for 30 min. (**L**) Immunostaining of iNOS and P-STAT1 in M1-polarized RAW264.7 cells treated with 5-methoxyflavone alone or in combination with brusatol treatment for 24 h. (**M**-**P**) M2-polarized RAW264.7 cells were generated by a 24 h treatment with IL-4 at 20 ng/mL, and then were repolarized into M1 phenotypes by incubation with LPS/IFN-γ for another 24 h. Flow cytometry analysis of M1 marker (iNOS) and M2 marker (CD206) in repolarization of M2-polarized RAW264.7 cells treated with 5-methoxyflavone alone (M and N) or in combination with brusatol treatment (O and P) for 24 h. ^###^*P* < 0.001 versus the control group; ^ζζζ^*P* < 0.001 versus the M2-polarized RAW264.7 cell group; ^**^P < 0.01, ^***^P < 0.001 versus the M2-polarized RAW264.7 cells with LPS/IFN-γ stimulation group; ^δδ^*P* < 0.01 versus the 5-methoxyflavone treatment alone. (**Q**-**R**) Western blot analysis of P-STAT1 in repolarization of M2-polarized RAW264.7 cells with indicated concentration of 5-methoxyflavone treatment alone (Q) or with combinations of 5-methoxyflavone plus brusatol treatment (R) for 30 min. (**S**) Immunostaining of iNOS and P-STAT1 in repolarization of M2-polarized RAW264.7 cells treated with 5-methoxyflavone alone or in combination with brusatol treatment for 24 h

Macrophage polarization plays a crucial role in governing the outcome of LPS-induced ALI because of the unbalance of the M1/M2 phenotype that is involved in inflammation exacerbation or resolution [9, 29]. We therefore sought to clarify whether Nrf2 activation by 5-methoxyflavone could modulate M1/M2 macrophage polarization in response to LPS challenge. Firstly, we evaluate cytotoxic effects of 5-methoxyflavone on RAW264.7 cells by MTT assay. The results showed that RAW264.7 cells with 5-methoxyflavone (0 - 60 μM) incubation for 24 and 48 h did not significantly alter their viability (Fig. 5A), indicating that 5-methoxyflavone had no cytotoxic effects on RAW264.7 cells at a concentration up to 60 μM. Therefore, the concentrations of 5-methoxyflavone lower than 60 μM were selected for the following experiments. Next, the morphology examination showed that LPS-stimulated RAW264.7 cells exhibited spindle-shaped pseudopodia and an irregular shape, whereas 5-methoxyflavone treatment reduced these morphological changes (Fig. 5B). Next, western blotting and immunofluorescence demonstrated that 5-methoxyflavone could also increase expression of Nrf2 and HO-1, and promote Nrf2 nuclear translocation (Fig. 5C and D). And flow cytometry analysis showed that LPS/IFN-γ-stimulated RAW264.7 cells with 5-methoxyflavone treatment significantly down-regulated the expression of M1 marker (iNOS), suggesting that 5-methoxyflavone has the capacity to suppress LPS/IFN-γ-mediated M1 polarization of RAW264.7 cells (Fig. 5E and F). Moreover, Nrf2 inhibition by brusatol was found to reverse the inhibitory effects of 5-methoxyflavone on LPS/IFN-γ-induced M1 polarization of RAW264.7 cells (Fig. 5G and H). M1 polarization of macrophages was generated by LPS/IFN-γ-mediated STAT1 signaling activation and activation of Nrf2 signaling could suppress M1 polarization of macrophages [30, 31]. We therefore speculated that the inhibition of M1 polarization by 5-methoxyflavone was perhaps due to Nrf2 activation and blocked STAT1 signaling. We found that RAW264.7 cells with LPS/IFN-γ stimulation for 0.5 h triggered maximum P-STAT1 activation in RAW264.7 cells, but the activation of P-STAT1 was attenuated in the later time points (1, 2, 4 and 8 h) (Fig. 5I). Therefore, in the following study, we investigated the effects of 5-methoxyflavone on LPS/IFN-γ-mediated P-STAT1 activation in RAW264.7 cells with LPS/IFN-γ stimulation for 0.5 h. As expected, the activation of LPS/IFN-γ-induced STAT1 signaling in RAW264.7 cells was significantly reduced by 5-methoxyflavone, which was obviously reversed by combination treatment with 5-methoxyflavone and brusatol (Fig. 5J and K). Meanwhile, immunofluorescence results showed that the inhibitory effects of 5-methoxyflavone on LPS/IFN-γ-induced iNOS expression and STAT1 nuclear translocation were abolished by Nrf2 inhibition (Fig. 5L). M2 macrophages could be repolarized into M1 phenotype, which would lead to aggravate inflammatory diseases [29]. We then investigated whether 5-methoxyflavone could affect the repolarization of M2 macrophages via its Nrf2-activated effects. Interestingly, we found that the M2-polarized RAW264.7 cells induced by IL-4 failed to increase the expression of the LPS/IFN-γ-induced M1 marker (iNOS) by 5-methoxyflavone treatment (Fig. 5M and N). Moreover, the reduction of M2 marker (CD206) caused by LPS/IFN-γ stimulation was reversed by 5-methoxyflavone (Fig. 5M and N). However, the reduced effects of 5-methoxyflavone on LPS/IFN-γ-mediated expression of iNOS on M2 macrophages were abrogated by brusatol (Fig. 5O and P). Simultaneously, the suppressive effects of 5-methoxyflavone on LPS/IFN-γ-mediated the reduction of M2 marker (CD206) were abolished by brusatol (Fig. 5O and P). Furthermore, the inhibitory effects of 5-methoxyflavone on LPS/IFN-γ-induced STAT1 signaling in M2 macrophages were reversed by brusatol (Fig. 5Q and R). Similarly, immunofluorescence results demonstrated that the suppressive effects of 5-methoxyflavone on LPS/IFN-γ-induced iNOS expression and STAT1 nuclear translocation in M2-polarized RAW264.7 cells were abolished by brusatol (Fig. 5S). Together, these data demonstrated that 5-methoxyflavone blocked LPS/IFN-γ-induced M1 polarization of RAW264.7 and repolarization of M2 into an M1 phenotype, which is due to activation of Nrf2 signaling, and thereby decreased activation of STAT1 signaling.

### 5-Methoxyflavone inhibited LPS-mediated lung injury in vivo

**Fig. 6:**
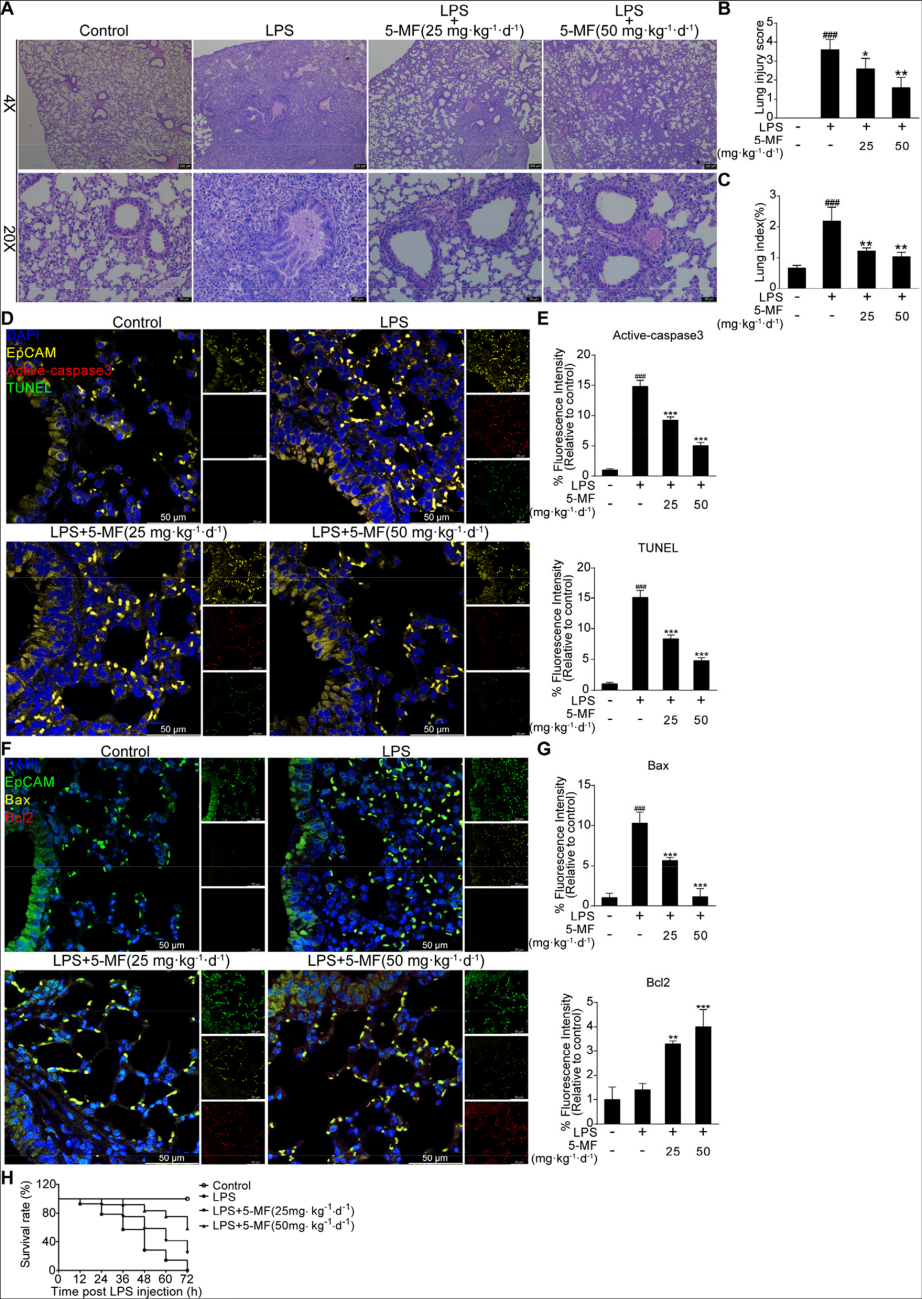
Protective effect of 5-methoxyflavone on LPS-induced ALI in mice. (**A**) Lung pathological changes of LPS-challenged mice with or without 5-methoxyflavone administration (i.g., 25, 50 mg⋅kg^-1^⋅d^-1^) were examined by H&E staining. (**B**) The lung injury score of all groups was assessed based on lung pathological changes. (**C**) Lung/body weight ratios (lung index; an indicator of lung injury) of all groups were measured at 48 h after LPS challenge. (**D**) Representative images of active-caspase 3 and TUNEL-positive cells in the epithelial (EpCAM^+^) cells of the lung tissues were presented. (**E**) The fluorescence intensities for active-caspase 3 and TUNEL in the epithelial (EpCAM^+^) cells were quantified. (**F**) Immunofluorescence staining was performed to assess the expression of Bax and Bcl2 in the epithelial (EpCAM^+^) cells of the lung tissues. (**G**) The fluorescence intensities for Bax and Bcl2 in the epithelial (EpCAM^+^) cells were quantified. (**H**) Survival curves for LPS-challenged mice with or without 5-methoxyflavone administration (*n* = 12 for each group). ^###^*P* < 0.01 versus the control group; ^*^P < 0.05, ^**^P < 0.01, ^***^P < 0.001 versus the LPS group

We further confirmed the protective effect of 5-methoxyflavone against LPS-mediated lung injury in vivo. Pathological analysis of lung tissue with H&E staining showed that interstitial inflammatory cell infiltration, bronchial epithelial sloughing and alveolar edema occurred in LPS-challenged mice, while 5-methoxyflavone administration markedly attenuated these pathological changes (Fig. 6A). Accordingly, the increased lung injury score caused by the LPS challenge was significantly reduced by 5-methoxyflavone administration (Fig. 6B). Compared to the control group, LPS challenge alone caused a higher lung index (lung/body weight ratio), a parameter of lung injury, which was decreased by 5-methoxyflavone administration (Fig. 6C). TUNEL-positive signals were predominantly detected in the lungs of LPS-challenged mice, suggesting LPS challenge led to an increased number of apoptotic cells in the lungs; in contrast, lungs from mice with 5-methoxyflavone administration showed much fewer TUNEL-positive cells (Fig. 6D and E). Moreover, suppression of LPS-triggered apoptosis by 5-methoxyflavone was further confirmed by the detection of decreased levels of active-caspase 3 in the lung tissues by immunofluorescence (Fig. 6D and E). In addition, to further confirm the anti-apoptotic effect of 5-methoxyflavone in LPS-mediated lung injury, the expression levels of Bcl-2 family proteins were detected by immunofluorescence. As shown in Fig. 6F and G, higher levels of Bax and lower levels of Bcl-2 were detected in the lungs of LPS-challenged mice than those of normal mice, whereas 5-methoxyflavone administration significantly increased the expression levels of Bcl-2 and reduced Bax expression levels. Finally, mice with an LPS challenge presented 100% mortality within 72 h. Remarkably, mice with 25 and 50 mg⋅kg^-1^⋅d^-1^ of 5-methoxyflavone administration increased the 72-h survival rate to 25% (3/12) and 58.3% (7/12), respectively (Fig. 6H). Therefore, these results indicated that 5-methoxyflavone could protect against LPS-mediated lung injury in vivo.

### 5-Methoxyflavone inhibited LPS-induced lung inflammation in vivo

**Fig. 7:**
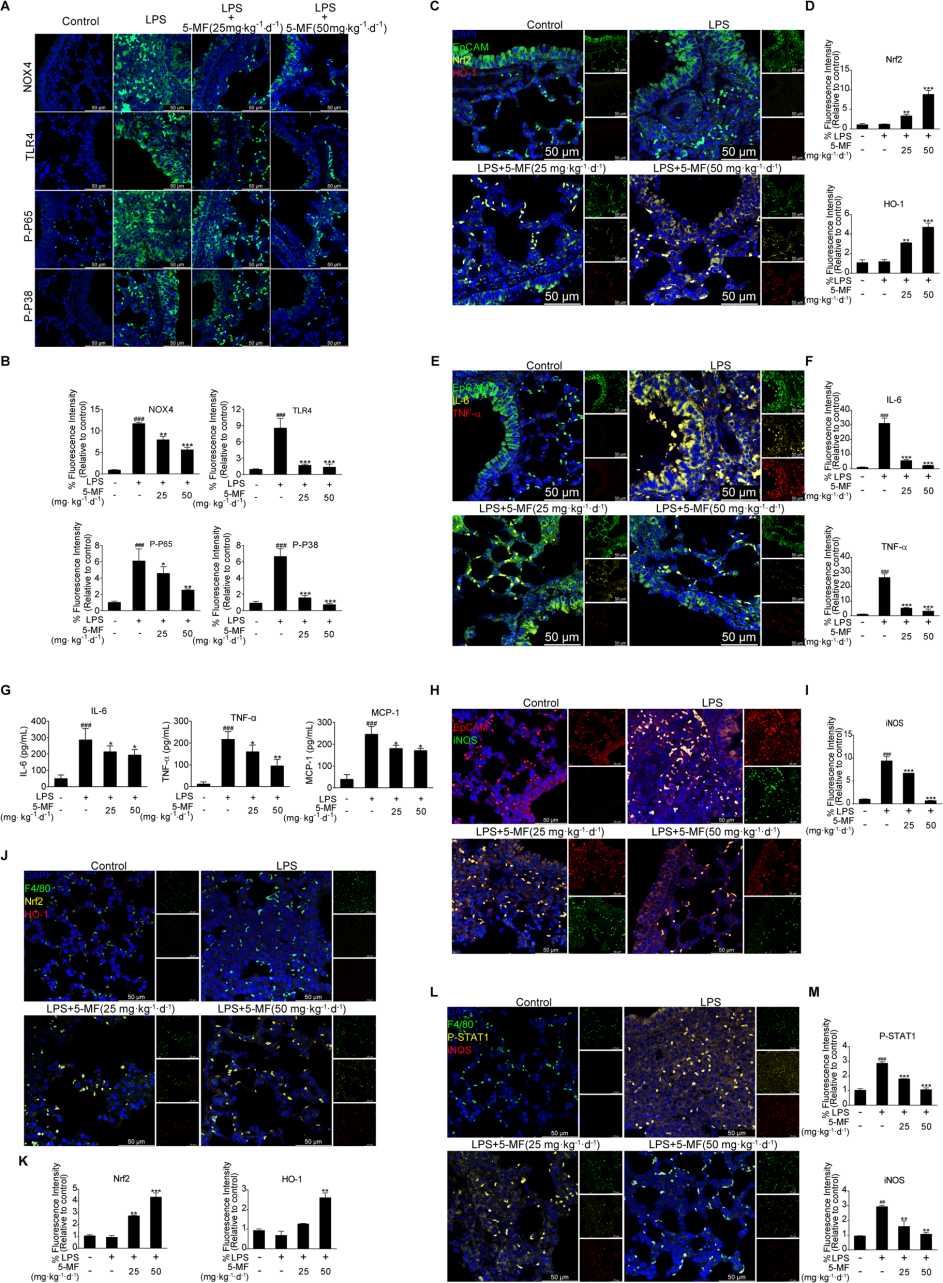
Effects of 5-methoxyflavone on LPS-induced lung inflammation in vivo. (**A**) Immunofluorescence staining was performed to assess the expression of NOX4, TLR4, NF-κB and P-P38 in the lung tissues. (**B**) The fluorescence intensities for NOX4, TLR4, NF-κB and P-P38 were quantified. (**C**) Immunofluorescence staining was performed to assess the expression of Nrf2 and HO-1 in the epithelial (EpCAM^+^) cells of the lung tissues. (**D**) The fluorescence intensities for Nrf2 and HO-1 in the epithelial (EpCAM^+^) cells were quantified. (**E**) Immunofluorescence staining was performed to assess the expression of IL-6 and TNF-α in the epithelial (EpCAM^+^) cells of the lung tissues. (**F**) The fluorescence intensities for IL-6 and TNF-α in the epithelial (EpCAM^+^) cells were quantified. (**G**) Measurement of pro-inflammatory mediators (IL-6, TNF-α and MCP-1) in the lung homogenates by ELISA assay. (**H**) Immunofluorescence staining was performed to assess the expression of iNOS in the epithelial (EpCAM^+^) cells of the lung tissues. (**I**) The fluorescence intensity for iNOS in the epithelial (EpCAM^+^) cells was quantified. (**J**) Immunofluorescence staining was performed to assess the expression of Nrf2 and HO-1 in the macrophage (F4/80^+^) cells of the lung tissues. (**K**) The fluorescence intensities for Nrf2 and HO-1 in the macrophage (F4/80^+^) cells were quantified. (**L**) Immunofluorescence staining was performed to assess the expression of F4/80, iNOS and P-STAT1 in the lung tissues. (**M**) The fluorescence intensities for F4/80, iNOS and P-STAT1 in the macrophage (F4/80^+^) cells were quantified. ^##^*P* < 0.01, ^###^*P* < 0.001 versus the control group; ^*^P < 0.05, ^**^P < 0.01, ^***^P < 0.001 versus the LPS group

The above results in Fig. 3D demonstrated that 5-methoxyflavone suppressed LPS-mediated NOX4/TLR4/NF-κB/P-P38 signaling through Nrf2 activation in vitro. We therefore employed immunofluorescence to examine the effects of 5-methoxyflavone on LPS-mediated NOX4/TLR4/NF-κB/P-P38 and Nrf2/HO-1 signaling in the airway and parenchyma of the lungs. Our results revealed that LPS challenge induced the intense expression of NOX4, TLR4, NF-κB, and P-P38 throughout the bronchial epithelial cells and pneumocytes of the lungs, which were significantly attenuated by 5-methoxyflavone administration (Fig. 7A and B). Meanwhile, treatment with 5-methoxyflavone could markedly increase the expression of Nrf2 and HO-1 in lung epithelial (EpCAM^+^) cells compared with those of the LPS group (Fig. 7C and D). It has been believed that excessive inflammation in sepsis could cause severe lung damage [32]. To examine the potential effects of 5-methoxyflavone on inflammation in response to LPS, we thus performed immunofluorescence to detect the levels of inflammation in lung epithelial (EpCAM^+^) cells. We found that LPS-triggered the increased levels of pro-inflammatory cytokines (IL-6, TNF-α) in lung epithelial cells was decreased in the lung tissues of mice with 5-methoxyflavone treatment (Fig. 7E and F). Likewise, LPS-induced expression of pro-inflammatory cytokines (IL-6, TNF-α and MCP-1) in the lung homogenates was decreased in mice treated with 5-methoxyflavone (Fig. 7G). Moreover, the increased levels of iNOS in lung epithelial (EpCAM^+^) cells caused by LPS was also effectively reduced by 5-methoxyflavone treatment (Fig. 7H and I). Given that 5-methoxyflavone reversed M1 polarization or the repolarization of M2 into M1 cells via activation of Nrf2 signal in vitro, it was interesting to investigate whether 5-methoxyflavone affected macrophage polarization in vivo. Consistent with the in vitro data, our results showed that 5-methoxyflavone administration could also activate Nrf2 signaling in the macrophage (F4/80^+^) cells of the lung tissues (Fig. 7J and K). Furthermore, high levels of F4/80^+^/iNOS^+^ macrophages linked to increased P-STAT1 activation were found in the lungs of mice that had been exposed to LPS, which were significantly reduced by 5-methoxyflavone administration (Fig. 7L and M). Together, these data showed that 5-methoxyflavone attenuated LPS-mediated inflammation and M1 polarization of macrophages, which may be associated with activation of Nrf2 signaling in vivo.

## Discussion

ALI is a severe and progressive respiratory disorder that poses a great threat to human health, but effective therapeutic treatments are still lacking. In the current study, in vitro experiments and in vivo studies were conducted to investigate whether 5-methoxyflavone could provide beneficial effects on ALI. Our findings confirmed that 5-methoxyflavone administration significantly alleviated lung injury and improved survival in mice exposed to LPS inhalation. In vitro experiments revealed that the protective effects of 5-methoxyflavone effectively suppressed LPS-mediated NOX4/TLR4/NF-κB/P38 MAPK signaling, resulting from its promoting Nrf2 pathway signaling activation in BEAS-2B cells. Moreover, Nrf2 activation by 5-methoxyflavone treatment abrogated the M1 polarization of RAW264.7 cells induced by LPS/IFN-γ and the repolarization of M2-polarized RAW264.7 cells into M1 phenotypes through inhibition of P-STAT1 activation.

Firstly, our results showed that 5-methoxyflavone has the capacity to reduce LPS-mediated ROS generation in BEAS-2B cells. Mounting evidence has indicated that oxidative stress is an important contributing factor in the pathogenesis of various diseases, including ALI [26]. During ALI, excessive ROS generation contributes to the endothelial and epithelial barrier disruption, resulting in leakage of fluids into the pulmonary parenchyma and massive leukocyte (e.g., neutrophils and macrophage) infiltration, as well as robust pro-inflammatory mediator production such as IL-6, TNF-α and IL-8 [1, 33, 34]. The conserved transmembrane enzyme NADPH oxidase (NOX) is well-known for its function in controlling ROS generation. Among the seven NOX family members (NOX1-NOX5, DUOX1, and DUOX2), the expression of NOX4 was inducible in endothelial cells [35], alveolar epithelial cells [36], and bronchial epithelial cells [37] under various pathological conditions. It has been reported that NOX4 knockdown decreased sepsis-induced ROS production, which was accompanied by an alleviation of ALI [35]. The interaction of NOX4 with TLR4 was found to be essential for LPS-triggered ROS production and subsequent expression of pro-inflammatory mediators [27]. Although we did not investigate whether 5-methoxyflavone affected the interaction between NOX4 and TLR4, we found that 5-methoxyflavone exerted an inhibitory effect on the expression of TLR4 and NOX4 in LPS-stimulated BEAS-2B cells and in the lungs of LPS-challenged mice. Meanwhile, data obtained from in vitro and in vivo studies showed that the increased levels of NF-κB and P38 MAPK, along with pro-inflammatory mediators including IL-6, TNF-α, IL-8, and MCP-1, were significantly reduced by 5-methoxyflavone. Previous studies demonstrated that the TLR4 downstream signaling molecules, including NF-κB and P38 MAPK, were phosphorylated, which jointly facilitates pro-inflammatory responses, thus exacerbating LPS-induced lung injury [6]. Pharmacological inhibition of TLR4/NOX4 signal suppressed LPS-mediated NF-κB and P38 MAPK activation, which reduced the expression levels of pro-inflammatory mediators [38, 39]. Based on these evidences, we supposed that 5-methoxyflavone suppressed LPS-mediated TLR4/NOX4/ROS signaling to reduce NF-κB and P38 MAPK activation, which resulted in attenuation of the pro-inflammatory response.

Interestingly, our findings showed that 5-methoxyflavone treatment increased expression of Nrf2 and drove its transcriptional activity, which is well-known for its function in controlling the expression of a series of antioxidant and detoxifying enzymes that provide cytoprotective activities against deleterious insults [11, 40]. In LPS-challenged mice, genetic deficiency of Nrf2 contributed to accelerated morbidity and mortality [41, 42]. Accordingly, the pathogenesis responsible for the disease-promoting effects of Nrf2 deficiency has been linked to increased NOX expression, ROS generation, TLR4 surface tracking, and NF-κB activation, leading to the amplified expression levels of cytokines and chemokines [43]. Whereas, pharmacological activation of Nrf2 improved LPS-mediated lung injury via repression of NF-κB and MAPKs (P38 and ERK1/2) signalings [44]. Consistent with these findings, the inhibitory effects on LPS-mediated TLR4/NOX4/ROS/NF-κB/P38 MAPK signaling exerted by 5-methoxyflavone were also reversed by Nrf2 inhibition, effectively eliminating 5-methoxyflavone's anti-inflammatory effects. As a result, we hypothesized that 5-methoxyflavone's ability to protect against LPS-induced inflammation and lung injury was due to its activation of the Nrf2 signaling pathway.

It is widely recognized that M1 polarization of macrophages releases pro-inflammatory cytokines, and alternatively, M2 macrophages secrete anti-inflammatory cytokines for the resolution of inflammation [45, 46]. The balance of the M1/M2 phenotype is crucial in determining the outcome of lung injury [9]. It has been demonstrated that chemokine environments affect macrophage polarization between M1 and M2 phenotypes [29]. M1 macrophages are generated by microbial-derived products and/or IFN-γ stimulation via STAT1 pathway activation, resulting in the production of excessive pro-inflammatory mediators (IL-6, TNF-α, and IL-12) that disrupt endothelial and epithelial barrier integrity and cause tissue damage [47, 48]. By contrast, the anti-inflammatory cytokines (IL-10 and arginase1) were produced by M2 macrophages as a result of IL-4/IL-13-mediated STAT6 activation, which promotes the repair of damaged tissue [47]. Our results showed that the polarization of the M1-like phenotype with spindle-shaped morphology caused by LPS induction was improved by 5-methoxyflavone treatment. Indeed, we found that the polarization of M1 macrophages by LPS/IFN-γ was blocked by 5-methoxyflavone treatment, which was found to have an effect on STAT1 signaling. Previous studies revealed that M2-skewed macrophages are highly plastic and could be repolarized into an M1 phenotype [49], and the sustained M1 phenotype switch resulted in aggravating inflammatory and continuing tissue damage [9]. Therefore, blocking the repolarization of the M2 to M1 transition may be effective in reducing excessive inflammatory responses and disease progression. Our results showed that the M2 polarization phenotype fails to convert into M1 cells upon LPS/IFN-γ exposure in the presence of 5-methoxyflavone treatment. Accordingly, 5-methoxyflavone attenuated the activation of STAT1 in M2 cells with LPS/IFN-γ stimulation. Moreover, 5-methoxyflavone treatment also activated Nrf2 in RAW264.7 cells. And Nrf2 activation was found to be involved in the regulation of M1/M2 macrophage polarization during ALI/ARDS [50]. Nrf2 activator or its downstream target gene has the capacity to suppress STAT1 signaling [51, 52], leading to preventing M1 polarization or the conversion of M2 macrophage to M1. This is in line with our findings that blockade of Nrf2 reversed the inhibitory effects of 5-methoxyflavone on LPS/IFN-γ mediated STAT1 activation, as well as M1 polarization or the repolarization of M2 into M1 cells. Thus, our data suggested that the activation of Nrf2 signaling by 5-methoxyflavone treatment suppressed LPS/IFN-γ mediated STAT1 signaling, which resulted in reversion of M1 polarization or the conversion of macrophage phenotype switching from M2 to M1.

**Fig. 8:**
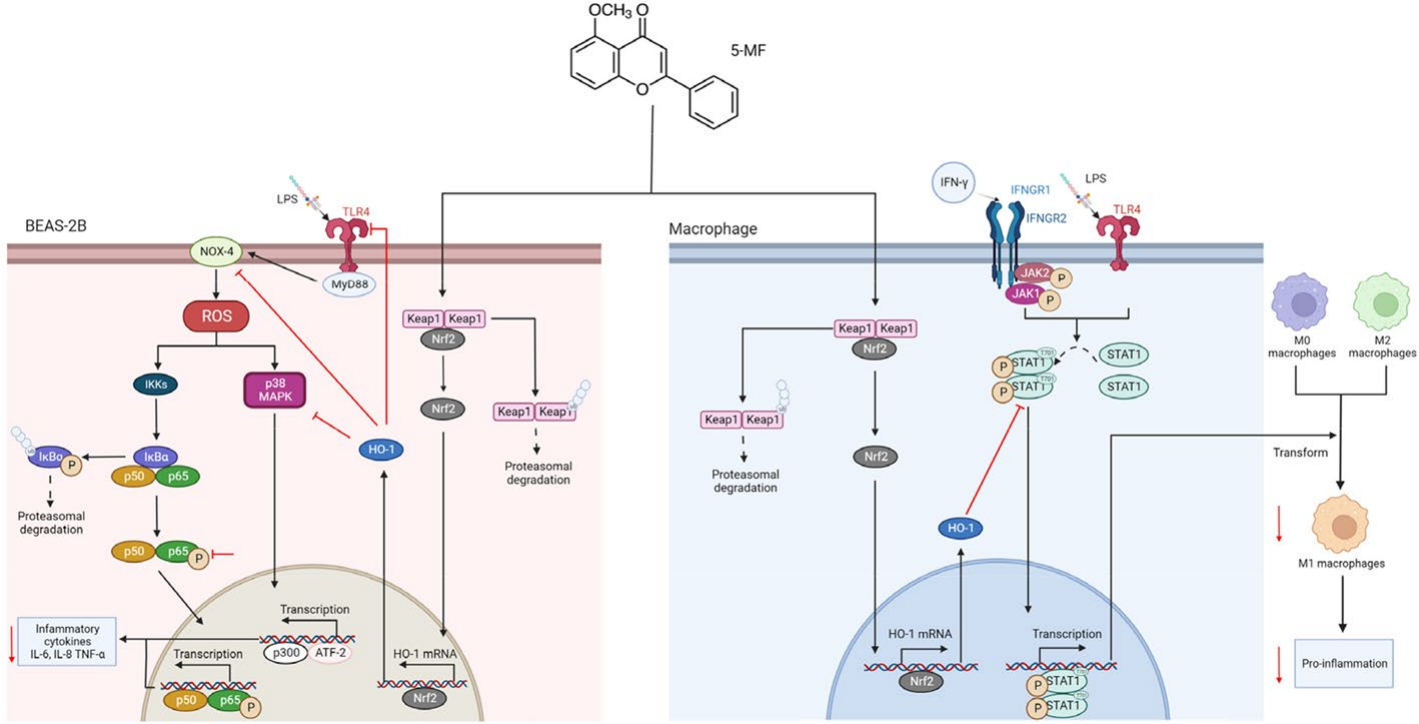
A proposed model illustrated the mechanism by which 5-methoxyflavone protected against LPS-induced lung injury. 5-Methoxyflavone has the capacity to activate Nrf2/HO-1 signaling, which resulted in attenuating the pro-inflammatory through suppression of TLR4/NOX4/ROS/NF-κB/P38 MAPK signaling in BEAS-2B cells. Furthermore, the activation of Nrf2/HO-1 signaling by 5-methoxyflavone inhibited activation of STAT1 signaling, and subsequently suppressed M1 macrophage polarization and repolarization of M2 macrophages into M1

In conclusion, our data suggested that activating Nrf2 with 5-methoxyflavone improved mice LPS-mediated lung injury and lethality by effectively suppressing the expression levels of pro-inflammatory factors via TLR4/NOX4/ROS/NF-κB/P38 MAPK signaling inhibition (Fig. 8). Furthermore, 5-methoxyflavone inhibited M1 macrophage polarization and the repolarization of the M2 to M1 phenotype through STAT1 signaling depends on its activated effects on Nrf2 (Fig. 8). Therefore, these findings suggest that 5-methoxyflavone could be a promising novel medicine candidate for ALI therapy.

## Data Availability

All data generated or analyzed during this study are included in this published article. The datasets used and/or analyzed during the current study are available from the corresponding author on reasonable request.
